# *Staphylococcus aureus* Biofilm Inhibiting Activity of Advanced Glycation Endproduct Crosslink Breaking and Glycation Inhibiting Compounds

**DOI:** 10.3390/antibiotics11101412

**Published:** 2022-10-14

**Authors:** Kyle Haasbroek, Masayuki Yagi, Yoshikazu Yonei

**Affiliations:** Anti-Aging Medical Research Center and Glycative Stress Research Center, Faculty of Life and Medical Sciences, Doshisha University, Kyoto 610-0394, Japan

**Keywords:** *Staphylococcus aureus*, biofilm, inhibition, glycation inhibition, AGE crosslink breaking, antimicrobial, glycative stress

## Abstract

*Staphylococcus aureus* is a Gram-positive bacterium that plays a role in the pathogenesis of skin lesions in diabetes mellitus, atopic dermatitis, and psoriasis, all of which are associated with elevated non-enzymatic glycation biomarkers. The production of biofilm protects resident bacteria from host immune defenses and antibiotic interventions, prolonging pathogen survival, and risking recurrence after treatment. Glycated proteins formed from keratin and glucose induce biofilm formation in *S. aureus*, promoting dysbiosis and increasing pathogenicity. In this study, several glycation-inhibiting and advanced glycation endproduct (AGE) crosslink-breaking compounds were assayed for their ability to inhibit glycated keratin-induced biofilm formation as preliminary screening for clinical testing candidates. Ascorbic acid, astaxanthin, clove extract, n-phenacylthiazolium bromide, and rosemary extract were examined in an in vitro static biofilm model with *S. aureus* strain ATCC 12600. Near complete biofilm inhibition was achieved with astaxanthin (ED_50_ = 0.060 mg/mL), clove extract (ED_50_ = 0.0087 mg/mL), n-phenacylthiazolium bromide (ED_50_ = 5.3 mg/mL), and rosemary extract (ED_50_ = 1.5 mg/mL). The dosage necessary for biofilm inhibition was not significantly correlated with growth inhibition (R^2^ = 0.055. *p* = 0.49). Anti-glycation and AGE breaking compounds with biofilm inhibitory activity are ideal candidates for treatment of *S. aureus* dysbiosis and skin infection that is associated with elevated skin glycation.

## 1. Introduction

Glycation is a nonenzymatic reaction that occurs in biological systems under physiological conditions between reducing sugars or aldehydes and proteins. After a series of reactions, advanced glycation endproducts (AGEs) are formed and accumulate throughout body tissues. AGEs trigger inflammation [1] and promote protein aggregation [2]. Glycative stress and its downstream effects contribute to the aging process and the etiology of disorders such as diabetes mellitus (DM) [3] and neurodegenerative diseases [4].

Glycative stress may also play a role in infectious disease by weakening immune functioning [5,6] and enhancing bacterial infiltration, as reported in DM associated urinary tract infection [7]. AGEs accumulate in the skin where they and can be measured non-invasively, acting as a biomarker for overall glycative stress and other factors such as arterial stiffness [8]. The glycation of the skin contributes to the appearance of aging and disrupts the skin’s barrier function [9]. AGEs are significantly elevated in inflammatory skin conditions atopic dermatitis (AD) [10,11] and psoriasis [12,13]. These diseases are characterized by the formation of skin lesions that are frequently colonized by pathogenic *Staphylococcus aureus*, further eroding the skin, exacerbating symptoms, and risking systemic infection. *S. aureus* and its antibiotic resistant variants are more frequently carried and in higher in abundance on the skin of those with AD [14], psoriasis [15], and DM [16]. 

It is speculated that the accumulation of AGEs in the epidermis may play a role both in the etiology of disorders such as AD and dysbiosis of the skin microbiome. It has been reported that glycated proteins enhance *S. aureus* biofilm formation [17,18], which may increase its potential for pathogenicity. Biofilm confers antibiotic resistance [19], protects resident bacteria from host immune responses such as phagocytosis [20], and perpetuates the dominance of *S. aureus* on AD skin [21]. Biofilm propensity of *S. aureus* strains is also correlated with increased severity of AD symptoms [22]. Eliminating *S. aureus* biofilms while combating the underlying glycative stress that contributes to skin dysfunction in AD, psoriasis, and DM is crucial for the prevention of recurring *S. aureus* skin and soft tissue infection and sustainable use of antibiotics. 

Glycative stress can be reduced with lifestyle and dietary choices that reduce blood sugar spikes [23,24]. Nevertheless, AGEs inevitably accumulate in the body over time with age, and once formed, are difficult to remove. Active interventions to breakdown AGEs and increase their clearance are being explored; numerous compounds have been discovered which demonstrate the ability to chemically cleave AGE-protein crosslinks or inhibit the glycation reaction [25]. Some of these compounds have shown promise in animal models [26], and see some use in the dietary supplement market. The applications of AGE crosslink breakers in topical skin treatments to reduce glycated protein accumulation have not yet been thoroughly tested. AGE breaking compounds may prove effective in cosmetic and medicinal skincare in treatment and prevention of skin dysfunction involving elevated glycative stress such as diabetic skin lesions. At the same time, *S. aureus* dysbiosis and biofilm formation is also a promising target: in addition to the breakdown of biofilm inducing AGEs, these materials may possess antimicrobial and biofilm inhibiting properties, making them ideal for treatment of *S. aureus* colonized skin lesions associated with glycative stress. 

In this study, several compounds with reported AGE crosslink breaking or glycation inhibiting activity were screened for their antimicrobial activity and ability to inhibit *S. aureus* glycated keratin-induced static biofilm formation.

## 2. Results

### 2.1. Biofilm Inhibition

Ascorbic acid, astaxanthin, clove extract, n-phenacylthiazolium bromide (PTB), and rosemary extract were tested. The results are summarized in Table 1. While ascorbic acid failed to completely inhibit glucose-keratin induced biofilm formation, all other compounds demonstrated efficacy at preventing biofilm formation, with clove exhibiting the strongest activity with an ED_50_ of 0.0087 mg/mL.

**Figure 1:**
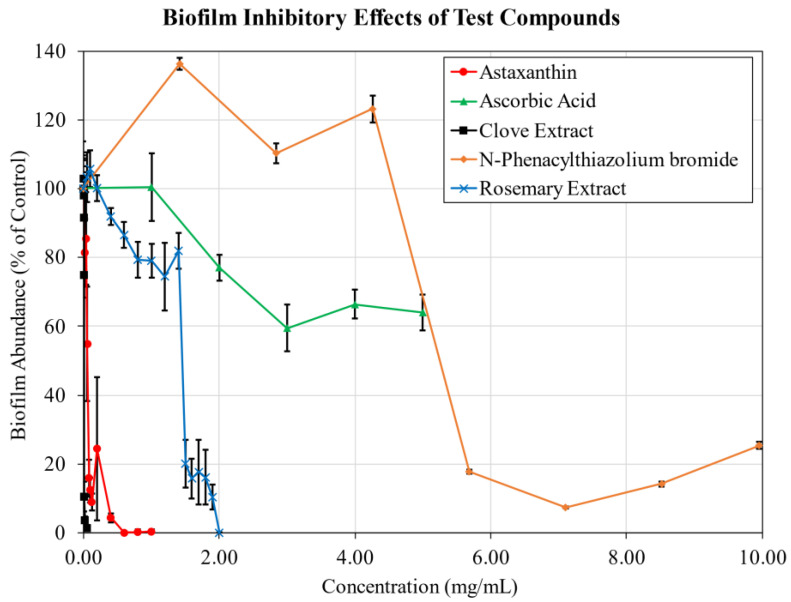
Biofilm Inhibition. Effect of test compounds on *S. aureus* biofilm formation provoked by 0.5 mg/mL of glucose-keratin. Biofilm assayed after 48 h of growth under static conditions. n = 8. Mean ± standard deviation.

Under static conditions *S. aureus* was incubated with 0.5 mg/mL of glycated keratin to induce a biofilm response, and the inhibitory activities of known AGE crosslink breakers and glycation inhibitors was assayed over a range of dosages (Figure 1). Negative control samples without glucose-keratin were also examined to determine the baseline effect on biofilm formation of each additive (Appendix A Figure A3). *S. aureus* ATCC 12600 does not produce substantial amounts of biofilm under standard conditions. When treated with glucose-keratin, biofilm production is stimulated in a dose dependent manner. Supplementation with 0.5 mg/mL glucose-keratin results in biofilms that fully covered the surface of sample wells, reaching absorbance values of roughly 1.7–2.0 after staining and solubilization. Typical absorbance values of control samples without glucose-keratin range from approximately 0.07 to 0.10; biofilm formation was considered fully inhibited when absorbance values were reduced to equal or lower than that of vehicle only control samples.

Astaxanthin, clove extract, and rosemary extract all significantly inhibited biofilm formation in a dose-dependent manner. Astaxanthin strongly reduced biofilm abundance at a low dosage, with an ED_50_ of 0.062 mg/mL and achieving total inhibition by 0.60 mg/mL. Clove extract demonstrated a stronger effect, with an ED_50_ of 8.7 µg/mL and achieving 98.6% inhibition by 0.050 mg/mL. Rosemary extract was effective at preventing biofilm formation at higher dosages; total biofilm inhibition was achieved at 2.0 mg/mL, with an ED_50_ of 1.5 mg/mL.

The effect of PTB on biofilm formation was varied. At sub-inhibitory concentrations, biofilm formation significantly increased; the addition of PTB to *S. aureus* cultures without glycated keratin similarly produced a significant increase in biofilm formation at sub-inhibitory dosages. Only at concentrations above 5.7 mg/mL was biofilm formation reliably prevented, coinciding with a considerable reduction in viable CFUs.

Ascorbic acid performed relatively poorly in biofilm inhibition testing. While ascorbic acid reduced biofilm formation to a peak reduction of 40.5% at 3 mg/mL, the inhibitory effect weakened with further increases in concentration. Without glycated keratin, ascorbic acid dosage was positively correlated with an increase in biofilm formation, resulting in a three-fold increase in biofilm above vehicle-only controls at 5.0 mg/mL.

### 2.2. Growth Inhibition

**Figure 2:**
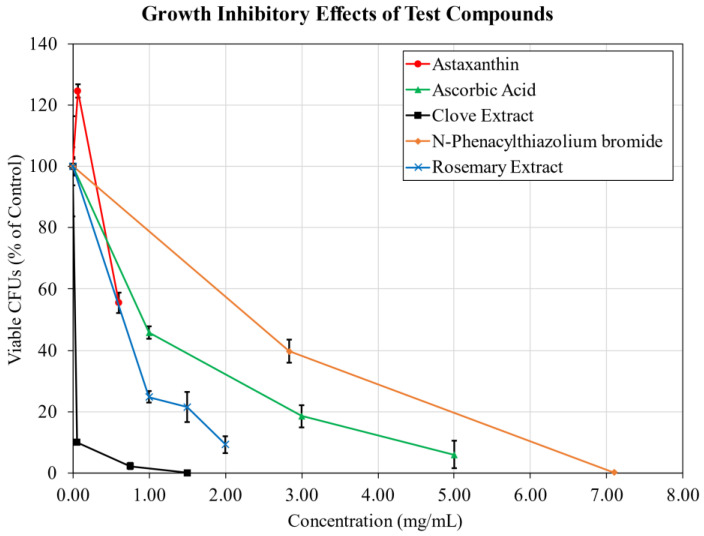
Growth Inhibition. Viable *S. aureus* CFUs after 24 h of incubation under planktonic conditions with additives. n = 3. Mean ± Standard Deviation.

In order to independently examine the growth inhibitory effects of the tested materials, growth cultures of *S. aureus* were supplemented with each of the compounds and incubated for 24 h before serial dilution and plate counting to determine the amount of viable colony forming units (Figure 2). Raw viability data for each material is shown in Appendix A Figure A2. The concentrations selected for testing were based on the results of the biofilm inhibition assays, targeting dosages approaching the observed ED_50_ and total biofilm inhibition if applicable.

Ascorbic acid, clove extract, and PTB demonstrated the strongest growth inhibitory effects of the tested compounds. Ascorbic acid reduced viable colony forming units (CFUs) in a dose dependent manner, reaching a log reduction of 1.2 at 5 mg/mL. Clove extract induced a 1.0 log reduction in viable CFUs at 0.050 mg/mL, increasing to a 3.4 log reduction at a concentration of 1.5 mg/mL coinciding with total biofilm inhibition. PTB at a sub-biofilm inhibitory dosage (2.8 mg/mL) weakly reduced CFU viability by 0.4 log, but achieved a 2.6 log reduction alongside substantial biofilm inhibition at 7.1 mg/mL.

The growth inhibitory effects of astaxanthin and rosemary extract were considerably weaker at the dosages examined. Astaxanthin viable CFU counts slightly increased compared to controls at 0.060 mg/mL, and were not substantially reduced at a near total biofilm inhibitory dose of 0.60 mg/mL. Rosemary extract significantly reduced growth in a dose-dependent manner, however the loss of viable CFUs did not exceed a 0.78 log reduction at the highest concentration of 2.0 mg/mL.

### 2.3. Correlation of Biofilm Inhibition and Growth Inhibition

**Figure 3:**
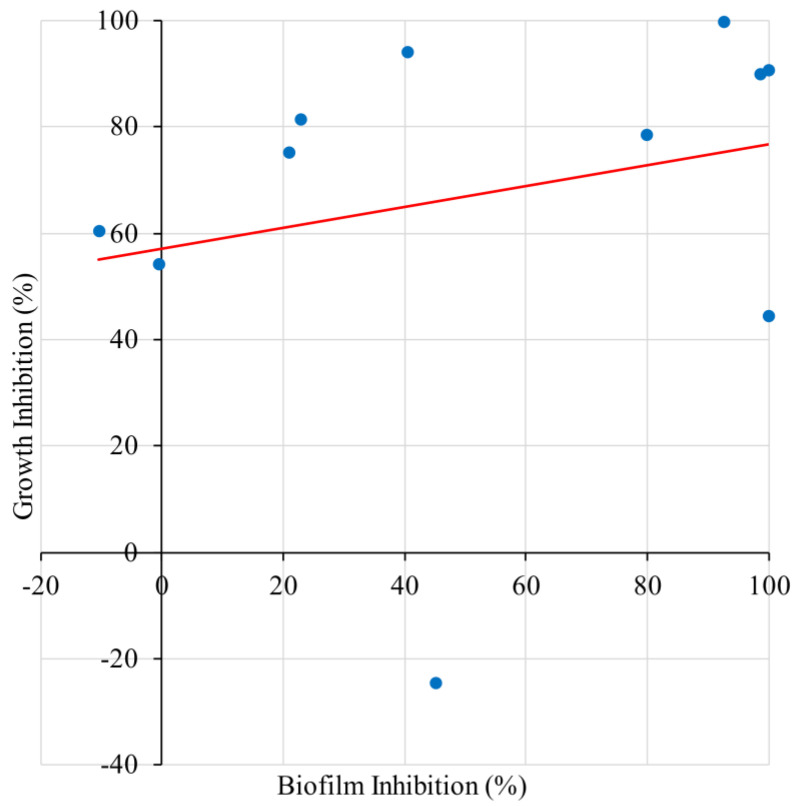
Relationship between biofilm inhibition and growth inhibition. Summary of collected data from cell viability assays and the corresponding biofilm inhibition at the same additive concentration, if available. n = 11. y = 0.20x + 57.13. R^2^ = 0.055. *p* = 0.49.

Summarizing the data collected from the cell viability assays and the corresponding biofilm inhibition results, a linear regression analysis was performed (Figure 3). Overall, biofilm inhibition did not correlate well with growth inhibition (R^2^ = 0.055, *p* = 0.49). All the tested materials demonstrated varying degrees of growth inhibition at sufficient concentrations. However, biofilm inhibitory activity was observed at sub-growth inhibitory dosages (with the exception of PTB), indicating that the antibiofilm activity of these compounds is stronger than the antibacterial. The mechanism of biofilm inhibition may also be independent of direct antibacterial activity.

## 3. Discussion

Ascorbic acid, commonly known as Vitamin C, is an essential dietary nutrient that is necessary for a variety of physiological processes in the human body, including wound repair and collagen production [27]. Ascorbic acid is also an antioxidant [28] with antibacterial activity [29], and the ability to disrupt exopolysaccharide biofilm formation in *E. coli* [30], *S. mutans* [31], *B. subtilis* [32], and *S. aureus* [33]. Ascorbic acid is a glycation inhibitor [25,34] via its antioxidant activity and ability to ionically bind to proteins, competitively inhibiting the binding of glucose.

Ascorbic acid appeared to be a strong candidate for treating biofilms: it has been reported [33] to prevent biofilm formation in methicillin resistant *S. aureus* by downregulating *ica*, consequently inhibiting Poly-β-1,6-*N*-acetyl-d-glucosamine (PNAG) production, the primary staphylococcal extracellular polysaccharide. In current testing, Ascorbic acid weakly inhibited biofilm formation at a dosage of 3 mg/mL, failing to completely inhibit biofilm formation as previously reported. This discrepancy suggests that AGE-induced biofilm formation may be *ica-*independent, which would explain the lack of efficacy. We have observed a significant increase in extracellular DNA released by *S. aureus* in response to glucose-keratin exposure (data pending publication), and it has been independently reported that *S. aureus* eDNA release regulators are promoted by AGE exposure [17]. Thus, the eDNA dominant biofilm phenotype may be more resistant to ascorbic acid. Strategies that are typically effective in the inhibition of primarily PNAG biofilms may be less effective in the treatment of AGE-related skin infections involving *S. aureus*.

Astaxanthin is a carotenoid produced by the algae *Haematococcus pluvialis*, and found in high concentrations in animals that consume the algae, such as salmon, shrimp, and crabs. Astaxanthin is a strong anti-oxidant [35] and anti-inflammatory compound, and is commercialized as a dietary supplement for these properties. Astaxanthin has also been found to exert anti-glycation effects [36,37]. Previous work in the lab has examined the effects of dietary astaxanthin intake in both animal and human models; astaxanthin was found to ameliorate high fat diet induced gut dysbiosis in mice, and improved reported gastro-intestinal symptoms in human participants [38].

While biofilm formations itself was inhibited, astaxanthin also appeared to have been incorporated into the biofilm itself, changing the white color of the unstained biofilm into a pale red. In addition to direct inhibition, the ability of the existing biofilm to adhere to the surface of the microwell plates was impaired. Remaining biofilm was easily detached by gentle washing, while patches of biofilm in the lowest dosage wells were strongly adhered and could not be removed even by vigorous shaking, as is typical of standard *S. aureus* biofilms under control conditions. When high concentration astaxanthin samples (0.6 mg/mL and higher) were examined, aggregation of *S. aureus* surrounding microscopic particles of astaxanthin was observed. This suggests that astaxanthin may be a competitive ligand for cell surface anchors, weakening surface attachment.

Astaxanthin is poorly soluble in water, therefore dimethyl sulfoxide (DMSO) was used as a solvent for in vitro testing. However, DMSO increases the permeability of the skin and causes skin irritation [39], thus it is not suitable for skincare use. Alternative solvents will be needed for clinical testing; the continued efficacy of astaxanthin in these formulations requires confirmation.

Cloves are produced from the dried flowers of the plant *Syzygium aromaticum*. Cloves are typically used as a culinary spice, but also have a centuries long history of use in traditional medicine for their anti-inflammatory properties. Modern research has demonstrated the powerful antioxidant [40] and anti-inflammation [41] activity of clove extracts. A significant active component of clove is eugenol [42], which has additionally demonstrated anti-carcinogenic/anti-tumor [43,44], and antimicrobial effects [45]. Unpublished data has revealed that extract from clove powder has powerful age cross link breaking activity, which is mediated by the compound biflorin. Considering these properties, the combined effects of eugenol and biflorin make clove extract a strong candidate for use in the AGE-biofilm system for potential treatment of *S. aureus* dysbiosis and associated skin lesions, although clove extract may also exert a cytotoxic effect on keratinocytes at sufficient dosages [46].

The crosslink breaking abilities of PTB have been demonstrated in both in vitro and in vivo studies [47]. PTB has shown effectiveness in breaking down AGEs and preventing their accumulation in vascular tissues [26], lens proteins [48], periodontic tissue [49] and bone [50]. No antibacterial or antibiofilm properties of PTB have not been reported at this time, although a significant growth inhibitory effect was observed at sufficiently high concentrations. Due to its biofilm enhancing effects at sub-inhibitory dosages, PTB may be less efficacious in antibiofilm applications.

*Salvia Rosmarinus*, known commonly as rosemary, is a plant that grows in the Mediterranean. The leaves of the plant have long been used as a culinary spice and in traditional medicines. Rosemary extract exhibits strong antioxidant and antibacterial effects [51] and is also known for its glycation inhibiting [52] and AGE cross-link breaking abilities [53] via its active ingredient rosmarinic acid. For this experiment, rosemary extract was utilized in the form of a dietary supplement named AGE Breaker (A2P Sciences, Lyon, France), marketed as an anti-aging supplement. While rosemary extract was effective at inhibiting glucose-keratin-induced biofilm formation, under control conditions without AGEs there was a dose-dependent increase in biofilm production (Appendix A Figure A3E). While the response was relatively small in magnitude, further testing is required to determine whether rosemary extract may be counter effective in practical applications where glycation may be lower than in vitro conditions.

## 4. Materials and Methods

### 4.1. Glycation Model

Glycated keratin solution used in testing was produced by incubating proteins with glycating agents in accordance with the recipes outlined by Hori et al. [54]. A solution comprised of 50 mM phosphate buffer (pH 7.4), 0.60 mg/mL keratin (Nacalai Tesque, Kyoto, Japan), and 40 mM glucose was prepared and incubated at 60 °C for 10 days. Ultrafiltration was performed using Amicon Ultra Centrifugal Filters according to the manufacturer’s instructions (Merck Millipore Ltd., Cork, Ireland) to remove unreacted glucose and concentrate protein above 10 kDA. Protein concentration was measured via Bicinchoninic Acid (BCA) Protein Assay (Thermo Fisher Scientific, Waltham, MA, USA).

### 4.2. Bacteria and Growth Conditions

*S. aureus* NBRC100910 (ATCC 12600) was purchased from the National Institute of Technology and Evaluation Biological Resource Center (Tokyo, Japan). Planktonic cultures were grown in Tryptic Soy Broth (TSB) at 37 °C, with 250 rpm shaking. Solid media cultures were grown on Tryptic Soy Agar at 37 °C. Growth media used was purchased from Becton, Dickinson, and Company (Franklin Lakes, NJ, USA).

### 4.3. Biofilm Inhibition Assay

**Figure 4:**
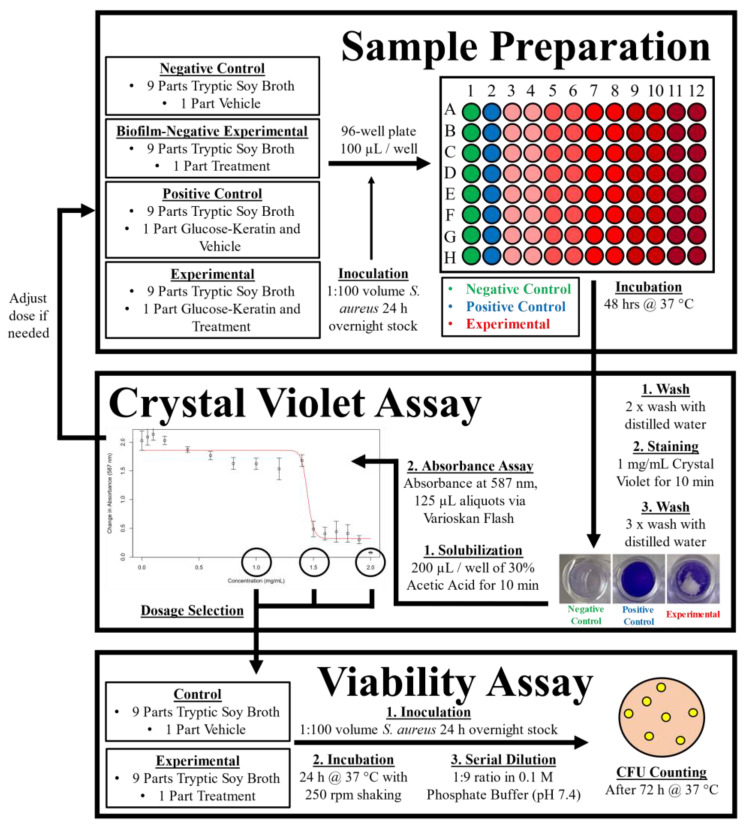
General Workflow Diagram.

The overall workflow of the experiment is presented in Figure 4.

Biofilm abundance was measured via crystal violet assay, modified from a widely used protocol [55,56,57]. Sample liquid cultures were prepared consisting of a 9:1 volume to volume ratio of TSB growth media and vehicle solution (ultrapure water or 5% DMSO in ultrapure water): a negative control was prepared without additives; a positive control contained 0.5 mg/mL of glucose-keratin; experimental conditions contained various concentrations of test compounds in the appropriate vehicle solution. Each solution was inoculated with 1:100 ratio volume (10 µL) of *Staphylococcus* overnight stock and thoroughly vortexed before transferring 100 µL aliquots into the wells of a sterile flatbottom 96-well plate (8 replications per sample). Plates were sealed and incubated at 37 °C for 48 h. Plates were twice washed with distilled water to remove unadhered bacteria before staining with 1.0 mg/mL crystal violet solution for 10 min. After staining, plates were gently washed with distilled water three times and air dried overnight. Biofilms were solubilized using 200 µL of 30% glacial acetic acid solution (Sigma-Aldrich, St. Louis, MO, USA). Finally, the absorbance of 125 µL aliquots at 587 nm was collected in triplicate using a Varioskan Flash Multimode Microplate Reader (Thermo Fisher Scientific, Waltham, MA, USA).

### 4.4. Cell Viability

Viability was determined by plate-counting after serial dilution in 0.1 M phosphate buffer (pH 7.4); Tryptic Soy Agar plates were inoculated with 100 µL of diluted culture media in triplicate and incubated at 37 °C for 72 h with daily colony counting.

### 4.5. Treatment Compounds

A list of the tested materials can be found in Table 2.

Ascorbic acid and *n*-phenacylthiazolium bromide were dissolved directly in ultrapure water at room temperature. 

A heated water extraction was performed for clove and rosemary extracts. For rosemary extract, a commercial anti-aging supplement, AGE Breaker was tested. Capsules of the supplement were dissolved in 40 mL of ultrapure water and heated to 60 °C for 60 min. Clove powder was solubilized in 40 mL of ultrapure water and heated to 80 °C for 75 min The resulting solutions were centrifuged at 2500 rpm for 10 min and the supernatant collected.

Astaxanthin was solubilized in DMSO at room temperature. For tests using astaxanthin all sample cultures were adjusted to a final DMSO concentration of 5.0 % (See Appendix A Figure A4 for DMSO data)

### 4.6. Logistic Regression Analysis

**Figure A1:**
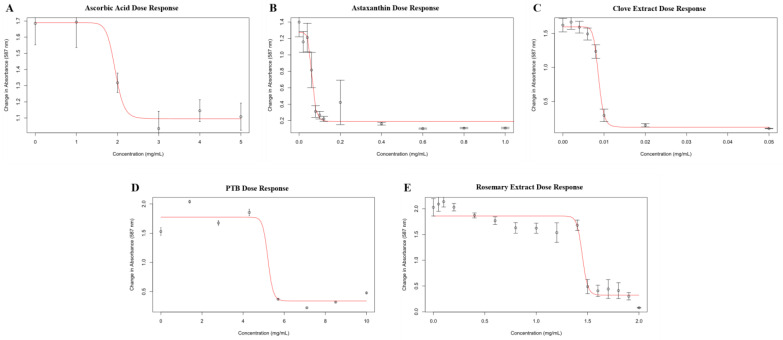
Biofilm Inhibition Logistic Regression. Effect of test compounds on *S. aureus* biofilm formation provoked by 0.5 mg/mL of glucose-keratin. (**A**) Ascorbic acid, R^2^ = 0.99. (**B**) Astaxanthin, R^2^ = 0.96. (**C**) Clove extract, R^2^ = 0.99. (**D**) PTB, R^2^ = 0.96. (**E**) Rosemary extract, R^2^ = 0.94. Biofilm assayed after 48 h of growth under static conditions. n = 8. Mean ± standard deviation. Logistic regression analysis performed using the dose–response curve package in R.

**Figure A2:**
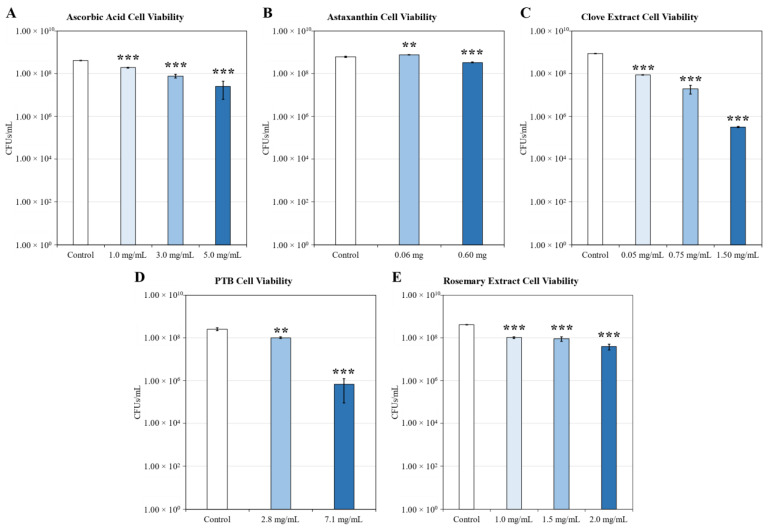
Cell Viability. *S. aureus* cell viability after 24 h of incubation under planktonic conditions with additives. (**A**) Ascorbic acid, (**B**) Astaxanthin, (**C**) Clove extract, (**D**) PTB, (**E**) Rosemary extract. n = 3. Mean ± Standard Deviation. Statistical difference determined by Student’s *t*-test compared to control. ** *p* <0.01, *** *p* < 0.001.

**Figure A3:**
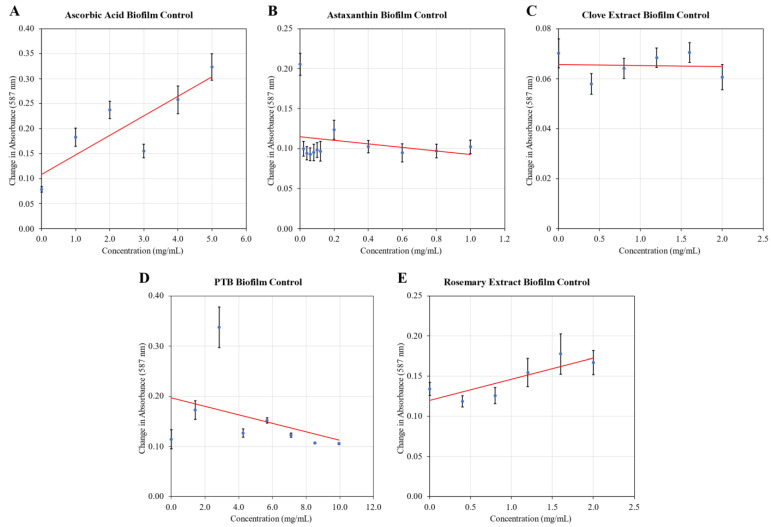
Effects of test compounds on biofilm formation in the absence of glucose-keratin induced biofilm formation. (**A**) Ascorbic acid: y =0.039x + 0.11, R^2^ = 0.72, *p* =0.32. (**B**) Astaxanthin: y = −0.0003x + 0.10, R^2^ = 0.0001, *p* = 0.97. (**C**) Clove extract: y = −0.0004x + 0.066, R^2^ = 0.0036, *p* = 0.91. (**D**) PTB: y −0.0085x + 0.20, R^2^ = 0.15, *p* = 0.35. (**E**) Rosemary extract: y = 0.026x + 0.12, R^2^ = 0.70, *p* = 0.039. n = 8. Mean ± standard deviation.

**Figure A4:**
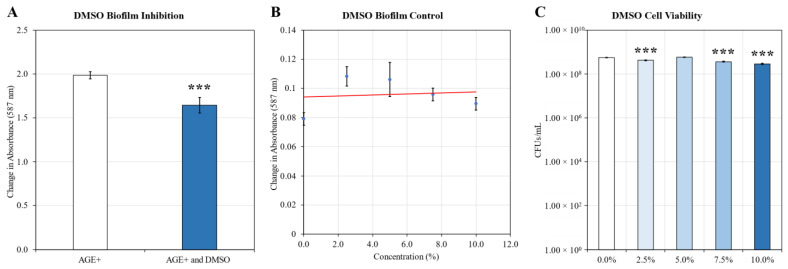
Effects of DMSO on biofilm formation and cell viability. (**A**) Biofilm inhibitory effect of 5.0% DMSO after 48 h of growth under static conditions with 0.5 mg/mL glucose-keratin. n = 8. Mean ± standard deviation. Statistical difference determined by Student’s t-test compared to control. *** *p* < 0.001. (**B**) Biofilm formation of DMSO alone after 48 h of growth. n = 8. Mean ± standard deviation. y = 0.0003x + 0.094, R^2^ = 0.012, *p* = 0.86. (**C**) *S. aureus* cell viability after 24 h of incubation under planktonic conditions with DMSO. n = 3. Mean ± Standard Deviation. Statistical difference determined by Student’s *t*-test compared to control. *** *p* < 0.001.

Logistic curve-fitting was performed in R [59] using the Dose Response Curve package [60] in order to determine the ED_50_ of each test compound with regard to biofilm inhibition. The results of curve fitting are shown in Appendix A Figure A1.

### 4.7. Other Statistical Analysis

Simple descriptive statistical analysis was performed using Excel 2016 version 2108 (Microsoft). Between group statistical difference was calculated using Student’s *t*-test. 

## 5. Conclusions

Astaxanthin, clove extract, and rosemary extract proved effective at inhibiting *S. aureus* biofilms induced by glucose-keratin. Clove extract was most powerful with an ED50 of 3.2 µg/mL. PTB inhibited biofilm only after reaching a critical concentration, and ascorbic acid was ineffective at completely preventing biofilm formation.

Further testing is necessary to evaluate the efficacy of AGE crosslink breaker clearance of glycated protein aggregates and reducing AGE accumulation in the context of topical application to the skin. Furthermore, the potential dysbiosis ameliorating effects of these compounds requires verification in beyond in vitro testing through in vivo and clinical study. If they continue to prove efficacious, compounds such as astaxanthin, clove extract, and rosemary extract may become a useful addition to the lineup of antimicrobial/antibiofilm treatments for combating *S. aureus* dysbiosis in skin disorder involving glycative stress, and the prevention of recurring infection of lesional skin in those with diabetes mellitus.

## Figures and Tables

**Table 1:** Summary of Biofilm Inhibition Results.

Material	ED_50_ (mg/mL)	Max Inhibition (% Control)
Ascorbic Acid	1.9	40.5
Astaxanthin	0.060	100.0
Clove Extract	0.0087	98.6
n-Phenacylthiazolium Bromide	5.3	92.6
Rosemary Extract (AGE Breaker)	1.5	100.0

**Table 2:** Reported Properties of Test Compounds.

Material	Manufacturer	Antimicrobial Activity	AGE Crosslink Breaking	Glycation Inhibition	Anti-Oxidant Activity
Ascorbic Acid	Wako Chemical (Osaka, Japan)	+ [31,32]	−	+ [25,34]	+ [28]
Astaxanthin	Sigma-Aldrich (St. Louis, MO, USA)	−	−	+ [36,37]	+ [35]
Clove Extract	House Foods (Osaka, Japan)	+ [43,45]	−	+ [58]	+ [40]
n-Phenacylthiazolium Bromide	Fluorochem (Hadfield, UK)	−	+ [26,48]	−	−
Rosemary Extract (AGE Breaker)	A2P Sciences (Lyon, France)	+ [51]	+ [53]	+ [52]	+ [51]

+: Effect is reported in the literature for the specified material. −: Publications regarding potential effects were not found during literature review.

## Data Availability

All data presented in this study are available upon request.
